# The absence of the caffeine synthase gene is involved in the naturally decaffeinated status of *Coffea humblotiana,* a wild species from Comoro archipelago

**DOI:** 10.1038/s41598-021-87419-0

**Published:** 2021-04-14

**Authors:** Nathalie Raharimalala, Stephane Rombauts, Andrew McCarthy, Andréa Garavito, Simon Orozco-Arias, Laurence Bellanger, Alexa Yadira Morales-Correa, Solène Froger, Stéphane Michaux, Victoria Berry, Sylviane Metairon, Coralie Fournier, Maud Lepelley, Lukas Mueller, Emmanuel Couturon, Perla Hamon, Jean-Jacques Rakotomalala, Patrick Descombes, Romain Guyot, Dominique Crouzillat

**Affiliations:** 1 Centre National de Recherche Appliquée au Développement Rural, BP 1444, 101 Ambatobe, Antananarivo Madagascar; 2 Department of Plant Biotechnology and Bioinformatics, Ghent University, Ghent, Belgium; 3 European Molecular Biology Laboratory, 71 Avenue des Martyrs, CS 90181, 38042 Grenoble Cedex 9, France; 4 Departamento de Ciencias Biológicas, Facultad de Ciencias Exactas y Naturales, Universidad de Caldas, Manizales, Colombia; 5 Department of Systems and Informatics, Universidad de Caldas, Manizales, Colombia; 6 Universidad Autónoma de Manizales, Manizales, Colombia; 7 Nestle Research, Société des Produits Nestlé SA, 1015 Lausanne, Switzerland; 8 Nestle Research-Plant Science Research Unit, BP 49716, 37097 Tours Cedex 2, France; 9 Boyce Thompson Institute for Plant Research, Cornell University, Ithaca, NY 14853 USA; 10 Institut de Recherche pour le Développement, UMR DIADE, Université de Montpellier, Montpellier, France; 11 VIB Center for Plant Systems Biology, 9052 Gent, Belgium; 12 Centro de Bioinformática y biología computacional de Colombia – BIOS, Ecoparque los Yarumos, Manizales, Caldas, Colombia; 13 Present Address: University of Geneva, CMU-Décanat, 1 Rue Michel Servet, 1211 Geneva 4, Switzerland

**Keywords:** Comparative genomics, Genome evolution, Plant genetics

## Abstract

Caffeine is the most consumed alkaloid stimulant in the world. It is synthesized through the activity of three known *N*-methyltransferase proteins. Here we are reporting on the 422-Mb chromosome-level assembly of the *Coffea humblotiana* genome, a wild and endangered, naturally caffeine-free, species from the Comoro archipelago. We predicted 32,874 genes and anchored 88.7% of the sequence onto the 11 chromosomes. Comparative analyses with the African Robusta coffee genome (*C. canephora*) revealed an extensive genome conservation, despite an estimated 11 million years of divergence and a broad diversity of genome sizes within the *Coffea* genus. In this genome, the absence of caffeine is likely due to the absence of the caffeine synthase gene which converts theobromine into caffeine through an illegitimate recombination mechanism. These findings pave the way for further characterization of caffeine-free species in the *Coffea* genus and will guide research towards naturally-decaffeinated coffee drinks for consumers.

## Introduction

**Figure 1:**
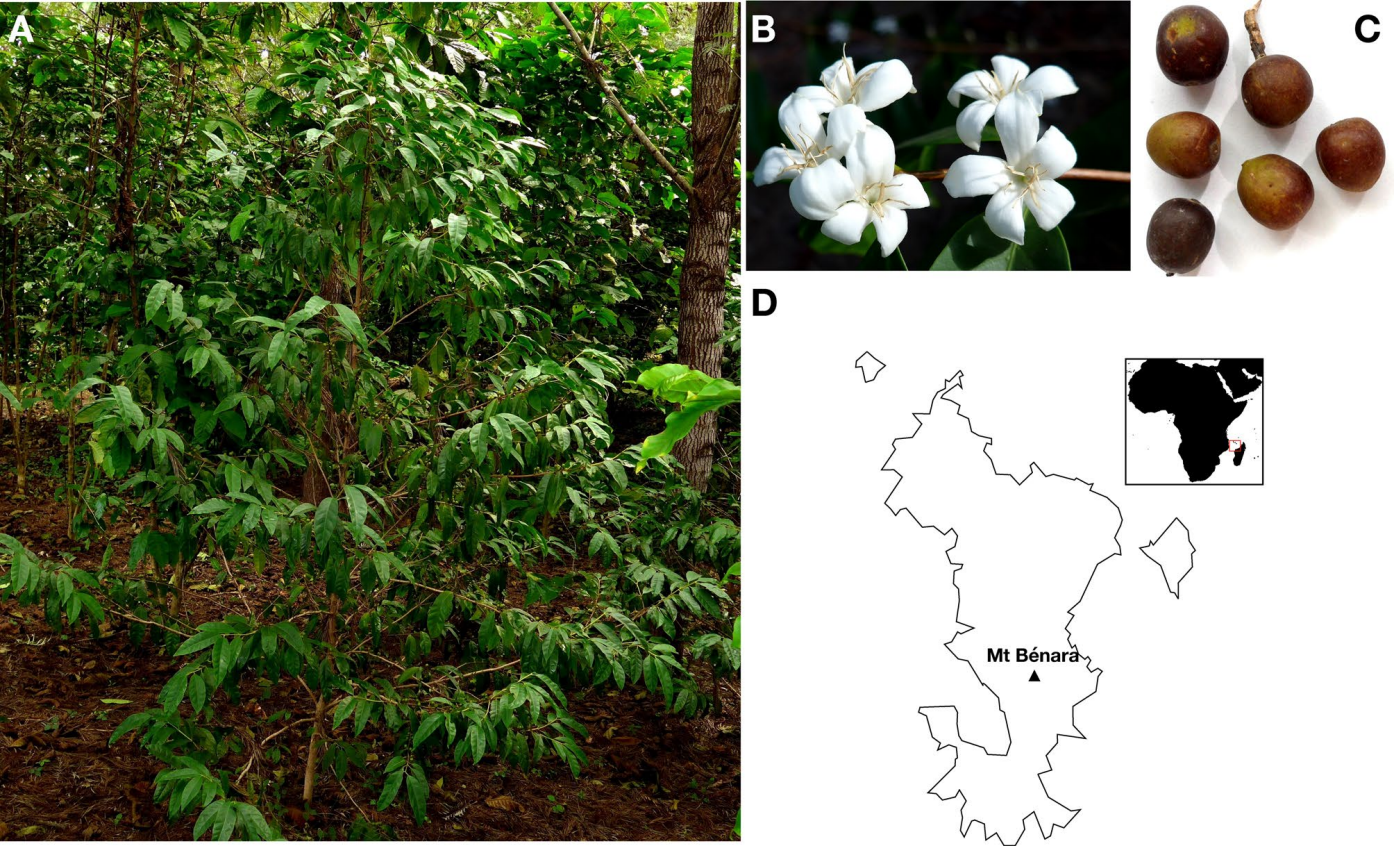
Representation of *Coffea humblotiana*. (**A**) Full tree of the *C. humblotiana* accession RM-CF-00679. (**B**) and (**C**) Inflorescences and collected fruits. Pictures were done by Emmanuel Couturon (IRD). (**D**) Location of Mayotte island. The map was drawn using Inkscape V.1.

*Coffea humblotiana* Baill., also called “*Caféier de Humblot*”, is the sole *Coffea* species endemic to the Comoro archipelago. It was probably consumed, even planted in the past on Grande Comore, a neighboring island of Mayotte in the archipelago, although the documentation on this subject remains very poor^1^. Due to local expansion of agricultural land, this species is now classified as endangered^2,3^. Currently, there are probably fewer than 110 trees surviving on Mayotte Island^3,4^ (https://www.iucnredlist.org) while its presence on the other islands of the archipelago remains unsure (Fig. 1). *C. humblotiana*, belongs to the *Coffea* genus, comprising 124 admitted species^3,5,6^, with a natural distribution covering tropical Africa, Madagascar, Comoros, Mauritius and the Reunion Islands, extending to southern and southeast Asia, and Australasia. It forms, with other Madagascan *Coffea* species, a large monophyletic clade separated from the African *Coffea* species an estimated 11.15 Mya^7^. All *Coffea* species are diploids with a chromosome number of x = 11, with the exception of *C. arabica,* which is an allotetraploid resulting from a recent cross between *C. eugenioides* and *C. canephora*^8^*.* The remarkable feature of *C. humblotiana*, is the complete absence of caffeine in seeds and leaves^9,10^, shared by most species from Madagascar, the Mascarene Islands and some species from East and Central Africa^7^. Caffeine is produced in the young leaves and immature fruits^11^ of African coffee species mainly, with a maximum level in *C. canephora*^10,12^ (2.4–3.3% dmb). The caffeine biosynthesis pathway involves three methylation steps catalyzed by different *N*-methyltransferase genes (*NMT*); the *XMT* gene (*xanthosine 7* N*-methyltransferase*), the *MXMT* gene (*7-methylxanthine methyltransferase*) and the *DXMT* gene^13,14^, (*3,7-dimethylxanthine methyltransferase* or *caffeine synthase*). The three *NMT* genes are located on two distinct regions in *C. canephora*, on chromosome 1 for the *DXMT* gene and on chromosome 9 for the *XMT* and *MXMT* genes^15^. As output trait for the industry, caffeine as well as chlorogenic acid (CGA) compounds are of a great interest since they participate in producing metabolites which activates five human bitter taste receptors, therefore contributing to an inferior final cup quality^16,17^. More particularly, the CGA degradation into phenol derivatives during roasting of coffee seeds contributes significantly to bitterness^18^.

In order to limit the amount of caffeine, different approaches have been followed such as RNA interference^19^ or interspecific crosses^20,21^, but with contrasting success. By investigating the natural variability of caffeine contents in *C. arabica,* three plants were identified with almost no caffeine^22^. Here, molecular analyses have suggested both a combination of transcriptional regulation and a mutation in the *DXMT* gene as likely responsible for the very low caffeine content of these mutants^23^, indicating that control of the caffeine synthesis pathway appears to be particularly complex^24^. However, as of yet, despite molecular investigations, no genomic characterization had been undertaken in naturally caffeine-free coffee species to discover the origin of this absence of caffeine, which limits any efforts to develop strategies for transferring this trait into the cultivated species.

Here we report the nearly complete assembly of the *Coffea humblotiana* genome, a wild endangered caffeine-free species from the Comoro archipelago, comprising 422 Mb of genomic sequences and 32,874 predicted genes. Comparative analyses with the African *C. canephora* (*C. canephora* Pierre ex A.Froehner) genome revealed extensive genome conservation, despite an estimated 11 million years of divergence and wide variation in genome size. We postulate here that it is the loss of the *Caffeine Synthase* (*DXMT*) gene (which converts theobromine into caffeine) through an illegitimate recombination mechanism, which is likely involved in the absence of caffeine. This loss corroborates with our findings of the presence of theobromine in the leaves. Our analyses encompass unprecedented information characterizing the genome of a wild caffeine-free species in the *Coffea* genus and bring forth a significant contribution towards developing a naturally-decaffeinated coffee drink.

## Results

### Genome sequencing, chromosome-level assembly and annotation

The size of the *C. humblotiana* genome was previously estimated to be 475 Mb using flow cytometry^25^. Based on 21 k-mer distribution of Illumina reads, a size of 406 Mb was predicted, with a heterozygous rate of 0.6% and a repeat frequency of 40% (Supplementary Figure S1). A de novo genome assembly was performed using 48 Gb of PacBio SMRT reads representing a coverage of about 102X (mean length = 7.6 Kb). This assembly produced 783 contigs for a total of 422 Mb with an N50 of 1.5 Mb (Table 1). To improve the contiguity of the assembly, Hi-C scaffolding was performed using 90 million paired-end reads of 150 bp. Finally, we obtained 390 scaffolds with a N50 of 29.6 Mb. The 11 largest scaffolds correspond to 88.7% (374.5 Mb) of the total size of the assembly and 92.5% of the k-mer-based genome size with pseudo-chromosome sizes ranging from 26,432,012 to 57,522,413 bp. Among the 390 scaffolds, 53 correspond to large and redundant fragments of the chloroplast genome, accounting all together for 2.8 Mb.

**Table 1:** Statistics for the *C. humblotiana* genome and gene annotation.

Number of scaffolds [#]	390
Total size of scaffolds [Mp]	420.72
Longest scaffold [bp]	57,522,413
N50 scaffold length [bp]	29,629,744
L50 scaffold count [#]	6
Number of genes	32,874
Average overall gene size [bp]	2,733
Average overall CDS size [bp]	1000
Average overall exon size [bp]	214

The BUSCO score revealed a completeness of 90.3% with only 88 missing genes. The *C. humblotiana* assembly was also evaluated for its contiguity by estimating the LTR Assembly Index (LAI) from LTR Retriever^26^. The *C. humblotiana* assembly shows a score of 10.73 indicating good contiguity of the assembly, while the *C. canephora* assembly has an LAI of 3.64, suggesting a greater contiguity for the *C. humblotiana* assembly as compared to *C. canephora* assembly. Finally, mapping genomic and transcriptomic Illumina reads on the final assembly using respectively Bowtie2^27^ and Hisat2^28^ produced an assembly completement estimate of 86.5–86.7% of alignment rates, while the RNA reads range between 86.8 and 88.6% (Supplementary Table S1). All estimations suggest good overall completeness of the assembly.

**Figure 2:**
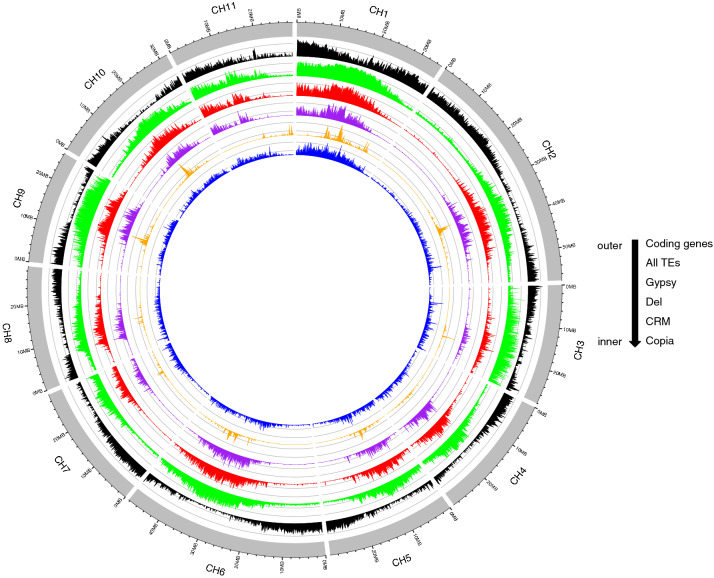
Features of the *C. humblotiana* genome. The density of the following features (predicted genes in black; all predicted transposable elements—TE—in green, Gypsy LTR retrotransposons in red; Del lineage, CRM lineage in orange and Copia LTR retrotransposons in blue) were calculated with a window’s length of 100 kb. Pseudochromosomes are oriented in a similar way to *C. canephora* available genome^15^.

The *C. humblotiana* genome comprised 32,874 predicted genes (Fig. 2, Table 1), relatively more than the *C. canephora* gene composition (25,574) (Supplementary Table S2). The proteomes from *C. canephora* (Denoeud et al.^15^ 25,574), Arabidopsis (27,910) and the predicted gene set from *C. humblotiana* (32,874) were compared using OrthoFinder. This platform assigned 59,113 genes (68.5% of total) to 16,350 orthogroups. Fifty percent of all the genes were in orthogroups of 3 or more genes (G50 was 3) and were contained in the 9761 largest orthogroups (O50 was 9761). There were 11,382 orthogroups with all species present and 7021 of these consisted entirely of single-copy genes (Supplementary Figure S2).

Transposable elements account for approximately 35% of the genome (143,851,980 bp). LTR retrotransposons Gypsy and Copia represent 19% and 4% respectively. The *Del* family represents 10.3% of the genome. Besides *Del*, *CRM* accounts for 3.5% and *Tork* 1.7%. This is significantly much lower than for the *C. canephora* genome for which almost 60% of the genome is represented by transposable elements, of which about 25% is represented by the Del family (Supplementary Figure S3). The diversity of LTR retrotransposons lineages was studied using reverse transcriptase (RT)-based phylogenetic analysis. We recovered 1992 RT domains from the sequenced genome that were aligned and analyzed. At the overall lineage level, all Copia and Gypsy elements observed in *C. canephora*^15^ were also present in *C. humblotiana* (Supplemental Figure S3, S4). In order to compare the difference in LTR retrotransposon content in detail, we performed an RT-based phylogenetic analysis grouping together recovered RT domains of *C. canephora* and *C. humblotiana*. The phylogenetic trees show major differences in the number of RT domains and LTR retrotransposon families of different lineages (Supplemental Figure S5). Specific amplification of *Del*, *TAT* and *CRM* for Gypsy and *SIRE*, *Tork* and *Bianca* for Copia was evident for *C. canephora* (in blue*,* Supplementary Figure S5), while little specific amplification could be observed for *C. humblotiana* (in orange). This observation suggests that the differential amplification of several LTR retrotransposon families in *C. canephora* may have occurred since the divergence of the two species, although some eliminations may also have occurred via unequal homologous and illegitimate recombination^29^.

The timing of LTR retrotransposon insertions was studied in the two genomes (Supplementary Figure S6). *C. canephora* and *C. humblotiana* genomes show different trends. A recent insertion of elements is observable between 0 and 1 My for *C. canephora* while a more gradual insertion of elements is visible between 0.5 and 5 My for *C. humblotiana*. In *C. canephora,* this activity is mainly due to Del, CRM, SIRE, Athila and TAT lineages. In *C. humblotiana*, most of the recent insertions are due to Del, while the insertion of CRM, Athila and TAT decrease in the last 0.5 My. Almost no recent insertion of SIRE is detected.

### Comparative genomic analysis of *C. humblotiana*

**Figure 3:**
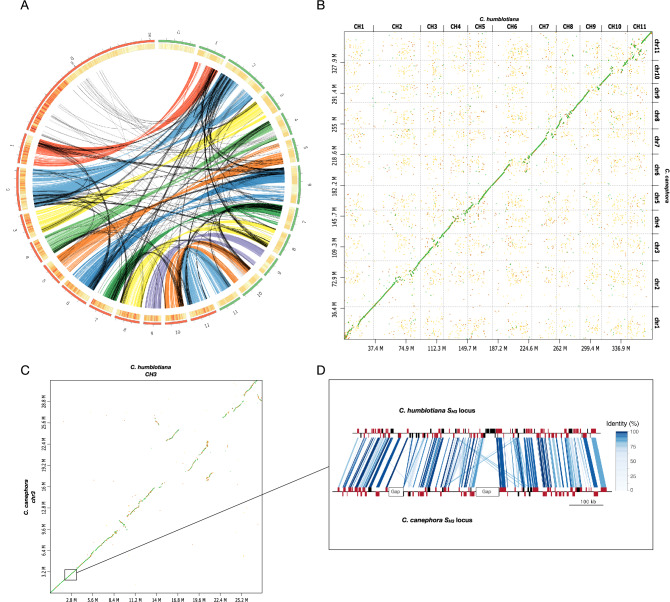
Comparative structural genomics between *C. humblotiana and C. canephora.* (**A**) Coding-region-based synteny between *C. canephora* (red) and *C. humblotiana* (green). For graphical purpose only, non-anchored contigs/scaffolds were merged into a single chromosome-zero (1000 N interspersed). (**B**) Whole genome dot plot between the *C. humblotiana* pseudo-chromosomes (horizontal sequence) and the *C. canephora* published pseudo-chromosomes (vertical sequence). (**C**) Dot plot between the pseudo chromosomes 3 of *C. humblotiana* and *C. canephora.* (**D**) Graphical representation of the 727 Kb region from *C. humblotiana* (upper line), and the 820 Kb region from *C. canephora* (lower line) at the S_H3_ locus, showing their annotated genes. Red boxes correspond to genes with one orthologous gene found in the compared segment, while black boxes account for unpaired or duplicated genes. White boxes represent a stretch of Ns found on the *C. canephora* genome. Colored lines linking both genomes represent the percentage of protein identity found in a pairwise comparison between genes.

The *C. humblotiana* and *C. canephora* assemblies were globally aligned using i-ADHoRe^30^ and D-genies^31^ (Fig. 3; Supplementary Figure S7). The overall chromosomal structure appears particularly well conserved at this level, suggesting a high degree of synteny, despite the lower quality of the *C. canephora* assembly^15^ (80% of the 710 Mb genome is assembled with an N50 scaffold of 26 Mb and only 51% of the scaffolds anchored onto pseudo-chromosomes). At the level of pseudo-chromosomes, conservation is restricted to the distal part of the sequences, while the putative centromere and pericentromeric regions showed few occurrences of conservation or fragmented ones (Fig. 3; Supplementary Figure S7). In order to make a comparison with a better-known genomic sequence of interest, the orthologous region bearing the Coffee Leaf Rust resistance locus *S*_*H3*_ in *C. liberica* (*C. liberica* W.Bull ex Hiern) and characterized in *C. arabica (C. arabica* L.), was retrieved from the reference (*C. canephora*) and from the *C. humblotiana* genomes, using the reported BAC clone sequences^32^ as a guide. A 727-Kb sequence comprised between positions 3,118,174 and 3,846,040 bp, and an 820-Kb sequence comprised between positions 3,208,179 and 4,028,477 bp were identified at the *S*_*H3*_ orthologous region and were extracted respectively from the *C. humblotiana* and the *C. canephora* genomes. A graphical comparison shows a very high collinearity of the two segments, with most genes having an orthologous pair and a high-protein identity, with the notable exception of the genes from *C. humblotiana* present in the corresponding *C. canephora* gaps (Fig. 3D).

### Evolution of *N*-methyltransferase genes related to caffeine synthase in *C. humblotiana*

By using BLAST analysis with *NMT* proteins related to caffeine synthase sequences annotated from *C. canephora*^13,15^ as queries against the *C. humblotiana-*annotated genome, we identified and manually inspected six *C. humblotiana* genes which were highly similar to these *NMTs* (Table 2; Supplementary Data S1; FASTA protein sequences of recovered genes). *NMT* genes were also searched and manually corrected in the assembled genome of *Gardenia jasminoides*^33^, (*Gardenia jasminoides* J.Ellis) a Rubiaceae species in the sub-family Ixoroideae^34^ for which only one complete gene was recovered: *Gj9*, similar to *GjNMT2* (Gj9A1032T108; pseudomolecule Gardenia 9). Two other genes were also located on pseudomolecule 1 (similar to GjA458T26 and GjA458T26), but precise manual annotation revealed stop codons and frameshifts for both of them. Besides the poorer quality of the genome sequence, one probable explanation is that these genes are probably non-functional.

**Table 2:** List of annotated and classified *NMT* genes.

Gene name	*C. canephora* gene ID	Chromosome	Putative orthologous gene ID in *C. humblotiana*	Chromosome
*NMT2*	cc02_g09350	Chr2	Cohum02g11490	Chr2
*DXMT*	cc01_g00720	Chr1	–	–
*XMT*	cc09_g06970	Chr9	Cohum09g08800	Chr9
*MXMT*	cc00_g24720	Chr9	Cohum09g08760	Chr9
*NMT3*	cc09_g06960	Chr9	Cohum09g08830; cohum09g08820	Chr9
*MTL*	cc09_g06950	Chr9	Cohum09g08730	Chr9

**Figure 4:**
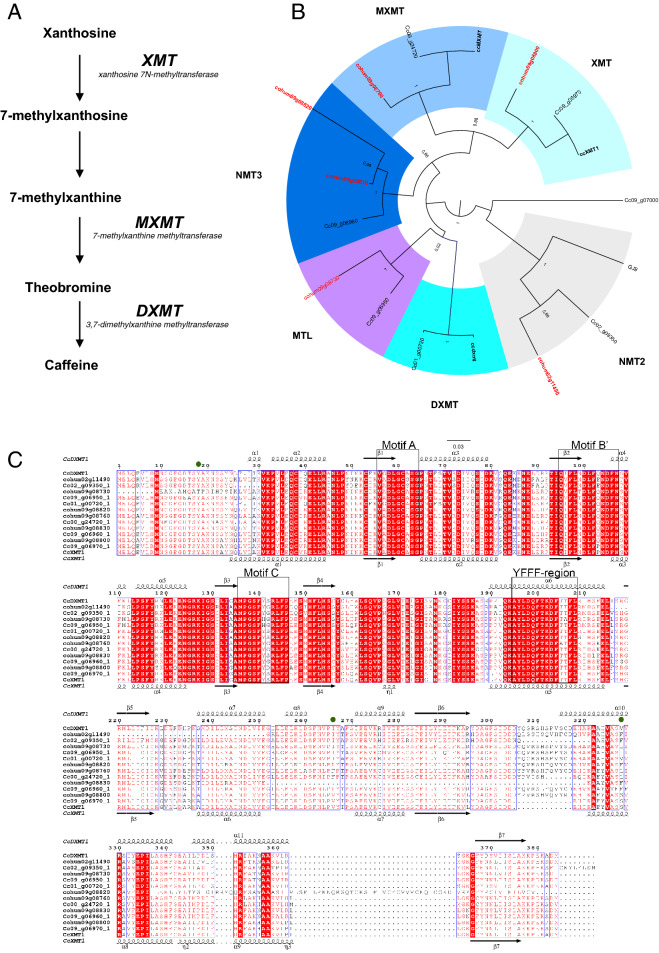
Evolution of *NMT* genes in *C. humblotiana* and *C. canephora.* (**A**) Representation of the methylation steps of the caffeine biosynthesis in coffee. (**B**) Phylogenetic analysis of complete NMT proteins in *C. humblotiana*, *C. canephora* and *Gardenia jasminoides*. Reference proteins for XMT (A4GE69), MXMT (jx978517) and DXMT (jx978516) are from *C. canephora*. IDs of *C. humblotiana* proteins are in red and IDs of *C. canephora* (in black) are named as in the genome annotation release. The protein Cc09_g07000 (NMT4) is used as outgroup. Numbers indicate the aLRT branch support. (**C**) Multiple sequence alignment of the *C. canephora* and *C. humblotiana MNT proteins.* Secondary structure plot is given for *CcDXMT* (CcDXMT (above) and CcXMT (below). The SAM binding motifs (A, B’ and C) and the conserved YFFF region are marked by boxes, and green circles identify crucial residues in substrate recognition and catalysis.

The protein sequences of all recovered genes were used for a phylogenetic analysis (Fig. 4A,B). It showed that each NMT protein in *C. canephora* was conserved in *C. humblotiana* (i.e. XMT, MXMT, MTL, NMT3 and NMT2) except for the DXMT protein. This gene is absent from the *C. humblotiana* assembled genome, and BLAST analysis against the sequence outputs of each step of the genome-assembly procedure failed to identify this gene. Except for DXMT, the phylogenetic analysis indicated that the NMT2 proteins assumed a basal position for the XMT, MXMT, MTL and NMT3 groups. Interestingly, *Gardenia* has only one complete NMT-like gene, *NMT2*.

It is peculiar to note how few RNA-seq reads from leaf tissue map to *XMT* and *MXMT* genes while numerous reads map to *MTL* in *C. humblotiana* (data not shown). This situation is different from *C. canephora* since XMT, MXMT and MTL were found highly transcribed in leaves^15^.

A sequence alignment of the *C. canephora* and *C. humblotiana* NMT proteins, and their comparison with the three-dimensional structures of *C. canephora* DXMT and XMT (PMID: 17434991) confirmed that all the genes identified contain the highly-conserved SAM binding motifs necessary for catalysis (Fig. 4C). However, only two of the *C. humblotiana* NMT proteins identified, XMT (cohum09g08800) and MXMT (cohum09g08760), are likely to be functional. The first NMT in the pathway, XMT (cohum09g08800), is clearly identifiable by the presence of a highly conserved serine (Ser-316 in CcXMT1) which is required for xanthosine recognition. The second NMT gene in the pathway, MXMT (cohum09g08760), can be annotated as an MXMT gene by the presence of a phenylalanine (Phe-266 in CcDXMT1), which prevents theobromine binding at the active site. The other four potential NMT genes are not likely to be involved in the caffeine biosynthesis. NMT2 (cohum02g11490) and NMT3 (cohum09g08830) are probably inactive due to large destabilizing deletions in their core protein fold, while NMT3 (cohum09g8820) contains a large and poorly conserved C-terminal extension that would also impact overall protein stability and activity. Lastly, the variable N-terminal sequence of MTL (cohum09g08730), compared with functional NMTs, and the absence of a highly-conserved tyrosine (Y18 in CcDXMT1) positioned adjacent to the active site and proposed to facilitate catalysis, both suggest that this NMT is not able to support methyl-transferase activity. To summarize, only two NMT proteins in the caffeine biosynthesis pathway of *C. humblotiana* are likely to be functional from a sequence comparison, suggesting that the biosynthesis pathway would stop at theobromine, corroborating the observed presence of theobromine.

**Figure 5:**
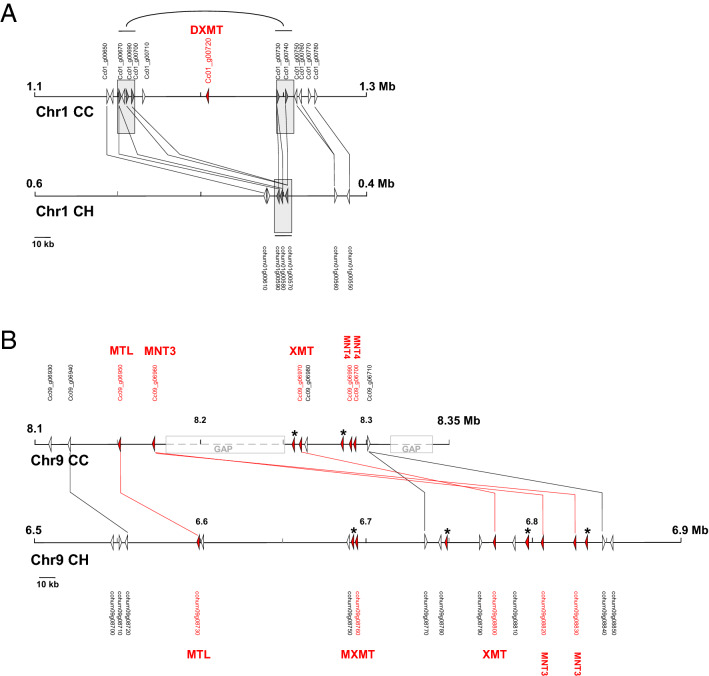
Microsynteny between *C. canephora* (CC) and *C. humblotiana* (CH) at the DXMT locus on chromosome 1 (**A**) and at the MXMT/XMT locus on chromosome 9 (**B**). (**A**) Representation of the microsynteny between *C. canephora* (CC; chromosome 1 position 1.1–1.3 Mb) and *C. humblotiana* (CH; chromosome 1 position 0.4–0.6 Mb). The *DMXT* gene, only present on *C. canephora* is indicated in red. The shaded boxes indicate the duplicated region in CC versus CH. (**B**) Representation of the microsynteny between *C. canephora* (CC; chromosome 9 position 8.1–8.35 Mb) and *C. humblotiana* (CH; chromosome 9 position 6.5–6.9 Mb). MTL genes are indicated in red. Asterisks indicate pseudogenes. In Grey are indicated the position of gaps in the assembly of the *C. canephora* genome. Triangles indicate the position and orientation of coding regions. Lines indicate the conservation of genes named by their IDs.

We conducted a microsynteny analysis between *C. canephora* and *C. humblotiana* to identify the evolutionary mechanisms that have shaped the regions of the *NMT* genes. In *C. canephora*, three loci carry the *DXMT, NMT2, XMT, MXMT, MTL* and *NMT3* genes on chromosomes 1, 2 and 9. The *DXMT* gene (*cc01_g00720*) is located on *C. canephora* chromosome 1 (positions 1,210,274–1,212,695) but it is absent in *C. humblotiana*. Microsynteny between the DXMT locus *C. canephora* (CC; the chromosome 1 position 1.1–1.3 Mb) and *C. humblotiana* (CH; chromosome 1 position 0.4–0.6 Mb) revealed the complete absence of a segment of 76 kb in *C. humblotiana* (Fig. 5). Interestingly, this segment carrying the *DXMT* gene in *C. canephora*, is flanked by a duplication of about 10 kb in *C. canephora*, while this region is present in only one copy in *C. humblotiana.* This observation suggests that the presence or absence of the *DXMT* locus might be due to either a large insertion (in *C. canephora*) or a deletion (in *C. humblotiana*) mechanism. However, the microsynteny between the *G. jasminoides* ortholog locus (Pseudochromosome 5, positions 2.2–2.5 Mb) and *C. canephora* also showed the insertion in *C. canephora* flanked by a duplication. Although the locus and gene are absent in *G. jasminoides*, the duplication is still kept in *G. jasminoides*, albeit degenerated. These observations may suggest several possible scenarios for the presence or absence of the DXMT locus, including an insertion in the ancestor of *C. canephora* after the *C. canephora*/*C. humblotiana* divergence, or an insertion in the ancestor of both *C. canephora* and *C. humblotiana* followed by a deletion in *C. humblotiana* (Supplementary Figure S8). Deletion in *C. humblotiana* could be due to a mechanism of illegitimate recombination (IR). IR produces a specific signature, with the presence of direct repeats flanking the duplicated regions or the presence of only one direct repeat associated with the deletion^35^.

A similar microsynteny analysis was performed at the MXMT/XMT locus on the *C. humblotiana* chromosome 9 (position 6.5–6.9 Mb). This locus is syntenic to the caffeine gene synthase cluster in *C. canephora* chromosome 9 (positions 8.1–8.35 Mb). However, the microsynteny is largely altered by numerous and different tandem duplications of *NMT* genes and insertion of transposable elements (Fig. 5). In the *C. humblotiana* MXMT/XMT locus, nine *NMT* genes constitute a cluster of tandem duplication of which four genes may be classified as pseudogenes. Pseudogenes correspond either to isolated exons of *NMT* genes lacking starting codons or to a complete gene (located at 6,789,033–6,795,587 bp) but displaying stop codons and a large insertion of a full-length LTR retrotransposon in the third intron of the pseudogene. The *NMT* tandem gene cluster in *C. humblotiana* is interrupted by frequent insertions of transposable elements. Moreover, the percentage of transposable elements reaches 63.4% in this region. The *C. canephora* syntenic regions follow the same trend with five *NMT* genes in tandem, two pseudogenes and 60.4% of transposable elements. It should be noted, however, that the *MXMT* gene in the *C. canephora* genome is not anchored to this locus but is still in an unanchored contig in the publicly available *C. canephora* genome assembly^15^. Furthermore, the *C. canephora* locus showed two large gaps due to anchoring difficulties during the genome-assembly step (Fig. 5). Recent improvements of the *C. canephora* genome assembly indicates the presence of the *MXMT* gene on the chromosome 9 locus (data not shown, personal communication).

The conservation of the NMT2 locus was also investigated, despite the fact that the NMT2 protein was not reported as participating in the caffeine-biosynthesis pathway. The *C. humblotiana* NMT2 locus (chromosome 2 position 7.5–7.7 Mb) was found highly syntenic with *C. canephora* (chromosome 2 position 7.2–7.4 Mb) (Supplementary Figure S9). In addition, a high degree of conservation was also observed between the *C. humblotiana* NMT2 locus and *G. jasminoides* chromosome 9 (positions: 103,200,000–103,300,000 bp)^33^ (Supplementary Figure S10).

### Biochemical analysis of caffeine and theobromine contents

Biochemical analyses were carried out on young leaves to characterize the chemical composition of the coffee species *C. canephora*, *C. arabica* and *C. humblotiana* using three different accessions for each. The results show significant contrasting quantities of caffeine and chlorogenic acids (CGA) among these three coffee species (Table 3 and Supplementary Table S3). The data confirms the absence of caffeine and the lower quantities of CGA in *C. humblotiana* (Table 3). These findings confirm previous studies based on biochemical analysis on seed^10^, which underlined that the biochemical diversity is related to the species analyzed. In this regard, the confirmed absence of caffeine in *C. humblotiana* was the main interest for our study.

**Table 3:** Caffeine, theobromine and total chlorogenic acids contents on young leaves of *C. arabica, C. canephora* and *C. humblotiana*.

	Caffeine			Theobromine			Total Chlorogenic acids		
	*C. arabica*	*C. canephora*	*C. humblotiana*	*C. arabica*	*C. canephora*	*C. humblotiana*	*C. arabica*	*C. canephora*	*C. humblotiana*
Minimum	0.91	2.16	0.00	0.01	0.00	0.04	4.14	5.04	0.40
Maximum	2.09	4.36	0.01	0.04	1.25	0.23	5.47	7.50	2.23
Means	1.50^a^	3.43^b^	0.003^c^	0.02^a^	0.42^ab^	0.13^b^	4.60^a^	5.89^a^	1.40^b^
Std dev	0.51	1.01	0.00	0.01	0.64	0.09	0.62	1.22	0.82

The biochemical compounds are average of three genotypes for each *Coffea* species (Supplementary Table S3). Data are expressed in percent of dry matter basis (% dmb) and their class membership, according to Kruskal–Wallis test, is indicated by a letter. All tests were significant (P < 0.001).

## Discussion

### The* C. humblotiana *genome is highly collinear with* C. canephora*

The *C. humblotiana* genome sequence reported here is the second published complete genome in the genus *Coffea* after *C. canephora*^15^ and third in the large Rubiaceae family (13,000 species^36^) after *Gardenia jasminioides*^33^. This assembly reveals overall good conservation of genomic collinearity with the African species *C. canephora*, despite an estimated genome divergence^7^ of more than 11 million years and a significant difference in genome-size. A small number of blocks show local inversions or rearrangements, but due to the different technologies used for genome sequencing and assembly, it is premature to conclude on the occurrence of these evolutionary events. A more complete assembly of *C. canephora* will be needed to allow a more precise synteny analysis. Whole genome duplications are expected to contribute to the evolution of gene function, but also promote genome modifications including gene losses and chromosome rearrangements^37^. This overall conservation of the collinearity between the two genomes is probably attributable to the absence of any recent whole genome duplication since the γ triplication at the origin of the Eudicots, in coffee trees and probably in Rubiaceae^15^, explaining its ancestral synteny with grapevine, two species that diverged 114–125 Mya^38^. The direct implication of the good conservation of the genomic collinearity is the ease of microcolinearity analyses in regions of interest between *C. canephora* and *C. humblotiana*, and a better understanding of their evolution and (re)organization over 11 My of divergence. We show that the *S*_*H3*_ region, which confers resistance to Coffee Leaf Rust^32^ (*Hemileia vastatrix*) in *Coffea liberica* and recently characterized in *C. arabica,* is strongly collinear in a syntenic block. This indicates that comparative genome analysis could provide crucial information in the evolution of the region, since the gene responsible for this resistance had not yet been characterized.

### LTR retrotransposons are mainly responsible of genome size variations

The genome size of *Coffea* species varies from 0.96 to 1.84 pg per 2C^39^. The smallest genomes come from East African species, Comoros and Indian Ocean islands, while the largest genomes are from West Africa and southeast Madagascar, suggesting gradients of genome size increase from East to West in Africa and North to Southeast in Madagascar^39^.

Previous studies, based on partial genome sequencing of eleven *Coffea* species, with 454 technology, have suggested the role of two LTR retrotransposon lineages, Del and Sire, in genome size variations and divergences^40^. However, the mechanisms underlying the variation could not be investigated due to the small amount of data produced. Here the comparison of the genome content in terms of transposable elements clearly indicates a higher number of LTR Retrotransposon of the Gypsy superfamily in *C. Canephora* with recent insertions of some TE lineages such as the Del, TAT, Athila and CRM. Our RT-based phylogenetic analysis highlights the expansion of these lineages that are accompanied by a diversification into new subfamilies for each lineage in *C. canephora*. Particularly, Del shows an interesting expansion in *C. canephora*, representing nearly 25% of the genome while only 10% in *C. humblotiana*. Interestingly, recent insertions of Del are also found in *C. humblotiana*, highlighting the activity of this TE lineage is accompanied by less accumulation of new copies, probably by mechanisms counter-acting its expansion. Of particular interest is the activity of the SIRE (Copia LTR retrotransposon) lineage. Showing few new insertions and few copies in *C. humblotiana*, SIRE demonstrates recent insertions and a diversification of new subfamilies in *C. canephora*. Preliminary studies have suggested that this lineage is virtually absent in Madagascar and Indian Ocean island *Coffea* species, marking its association mainly with the genomes of African *Coffea* species.

### The *C. humblotiana* genome also shed light on the evolution of genes involved in the caffeine biosynthesis pathway

Gene annotation and detailed microsynteny analyses with *C. canephora* revealed the absence of a segment of 76 kb containing the *DXMT* gene on chromosome 1 that converts theobromine to caffeine, probably due to an illegitimate recombination. This deletion is likely responsible for the lack of caffeine in *C. humblotiana* tissues, although it may also be the evolutionary consequence of a more complex mechanism. The absence of the caffeine synthase activity is supported by the detection of theobromine—the precursor of caffeine—in leaves, since the two first *NMT* genes located on the caffeine pathway (i.e. *XMT* and *MXMT*) appear functional, converting xanthosine to theobromine. However, in caffeine-free *C. arabica* mutants, higher levels of theobromine contents were detected in leaves^22^. Similarly, a high accumulation of theobromine was also detected in young shoots of Hongyacha, a naturally caffeine-free tea plant^41^. Unlike *C. humblotiana*, the *DXMT* gene is present in both *C. arabica* mutants and Hongyacha. In *C. arabica* mutants with low caffeine content, a mutation in the *DXMT* gene apparently dramatically reduces its caffeine synthase activity. A low enzymatic activity of caffeine synthase has even been detected in fruits, suggesting that some transcripts were able to be translated to functional proteins^24^. In Hongyacha, the *Tea* c*affeine synthase* gene (*TCS*) is unable to produce caffeine but keeps a reduced activity of theobromine synthase since the *TCS* gene has a dual activity, being able to add methyl groups on both 7-methyxanthosine and theobromine. One possible explanation for the reduced theobromine content in *C. humblotiana* leaves compared to *C. arabica* mutants leaves would be a similar dual-functional activity of the caffeine synthase protein as the TCS gene^42^. Hence, a dual activity of the caffeine synthase gene (*DXMT*) has been reported in *C. arabica*, where it is able to add methyl groups on both 7-methyxanthosine and theobromine. Therefore, the simple absence of the *DXMT* gene in the *C. humblotiana* genome would lead to both the reduction of the theobromine accumulation and the absence of caffeine, while the mutation of the *DXMT* gene in *C. arabica* would only lead to a partial loss of its function. Contradictory to these studies, Maluf and coworkers identified a decrease of theobromine synthase transcripts accumulation in different fruit stages of *C. arabica* mutants, hypothesizing a mechanism of feedback regulation activated by the accumulation of theobromine^23^. Such feedback regulation of the caffeine biochemical pathway was recently highlighted in tea leaves (*Camelia sinensis*) in which the pathway is fully functional^43^. In *C. humblotian*a a possible feedback regulation could participate to the reduction of theobromine accumulation since very few transcriptions of the *XMT* and *MXMT* genes was observable using leaves RNA-seq read mapping. Further analysis of co-expression of transcript/metabolic networks including theobromine degradation metabolites may be needed to decipher the complex regulation of the caffeine pathway in *C. humblotiana.*

Apart from the absence of the *DXMT* gene, there are few alternative hypotheses able to explain the absence of caffeine in *C. humblotiana.* In *Coffea millotii* J.-F.Leroy and *Coffea perrieri* Drake ex Jum. & H. Perrier, two *Mascarocoffea* species, the biosynthesis of purine alkaloid stops at the 7-methylxanthine step formation and both the theobromine synthase and caffeine synthase activities are missing^44^. These absences are accompanied by the reduction of the total purine alkaloid biosynthesis activity with a shift of purine metabolites toward purine catabolism. Similar results were observed with anti-sense and RNA interference transgenic plants of *C. canephora MXMT* gene^45^. A such initial shift in the purine metabolites pathway would have left the possibilities of subsequent accumulation of neutral mutations such as a deletion of the *DXMT* gene.

Based on our comparative genome data, we hypothesized that the absence of caffeine in *C. humblotiana* would derive from a direct ancestor that was capable of producing this alkaloid. This is intriguing, since the complete absence of caffeine is shared by geographical groups of the wild coffee species from Indian Ocean islands, East Africa and a group of ancestral species formally called the genus *Psilanthus,* suggesting that the ancestor of all coffee trees would be caffeine free^7,46^. However, some exceptions exist in geographical groups such as in Africa and in the Indian Ocean island, but not in the former Psilanthus genus. For example, the Central African *Coffea charrieriana* Stoff. & F.Anthony does not synthetize caffeine^47^. On the other hand, a very low content (0.07%) of caffeine was reported in *Coffea mauritiana* Lam. seeds^47^, and two species from Madagascar (*Coffea kianjavatensis* J.-F.Leroy and *Coffea lancifolia* A.Chev. subsp. *auriculata*) showed significant amounts of caffeine (0.55% and 0.81% dmb)^48^. These findings do not question the caffeine-free ancestor hypothesis at this stage, but may suggest more complex mechanisms to explain the absence of caffeine in coffee species in Africa and Madagascar and possibly different genetic causes. Further genomic studies in the *Coffea* genus are necessary to evaluate the presence of functional *NMT* genes. This will help to construct a robust hypothesis on the origin and timing of evolutionary mechanisms targeting *NMT* genes in caffeine-free *Coffea* species and build an evolutionary model.

The microcolinearity of the XMT/MXMT locus on chromosome 9 revealed a specific pattern of evolution linked to the *NMT* genes, with tandem duplication and pseudogenization of copy in a transposable element-rich environment (60%). The orthologous regions in the *G. jasminoides* genome^33^ show a comparable organization, suggesting a common pattern of evolution similar to the Birth-and-Death model of resistance-gene clusters (NBS-LRR type) in plants^49^. It involves unequal intergenic crossing-over mechanisms, generating losses and duplications as well as mutations that might explain genetic changes on chromosome 9 NMT regions. Similar to resistance gene clusters, *NMT* tandem gene copies are associated with a rich transposable-element environment.

In conclusion, the assembly *of C. humblotiana* generated in the frame of the present study provides the first high-quality reference genome for the *Coffea* genus. It provides valuable information for promoting the preservation of the diversity of this wild species in its environment and it represents a perfect resource for genomic and evolutionary studies on *Coffea* and *Rubiaceae*. It is also of interest in helping to develop new strategies for characterizing coffee cup-quality traits. However, additional and substantial efforts will be necessary to develop strategies for improving coffee cultivars. In this regard, the absence of caffeine and the low CGA content of *C. humblotiana* beans, which together strongly reduce the bitterness of coffee beverage, is an interesting avenue to follow. However, this species may contain another bitter compound: cafamarine (glycosidic diterpene)^50,51^, the content of which will need to be understood and controlled to open the door to new sensory experiences for consumers in the future.

## Methods

### Plant material and biochemical analysis

All the plant material used in this study is compliant with the Nagoya protocol and with the Access and Benefit-sharing Clearing-House (ABSCH-IRCC-FR-254781-1). Three genotypes were analyzed for each *Coffea* species (*C. arabica, C. canephora* and *C. humblotiana)*. For *C. arabica* we selected GPFA03, ET39 and GPFA107, for *C. canephora* the samples were coded as 16M, FRT95 and FRT141-8 and for *C. humblotiana* three plants from bulk seedlings were randomly chosen (Supplementary Table S3). Samples were growing in greenhouse. Samples from young leaves (2–3 weeks old) were ground in liquid nitrogen and 500 mg of the resulting powder were add to 70 ml of a solution of 70% methanol for 30 min at 40 °C. Caffeine, theobromine and chlorogenic acids (CGA) were analyzed using the Dionex HPLC U3000. Two mobile phases were used: (1) an aqueous solution containing 8% acetonitrile and 0.1% formic acid (Mobile Phase 1); and (2) an aqueous solution containing 50% acetonitrile and 0.1% formic acid (Mobile Phase 2). Samples and standard solutions (10 µl) were analyzed at 30 °C using a gradient elution with flow rate of 0.8 µl/min and UV detection at 272 nm and 325 nm wavelengths, corresponding to, respectively, caffeine and theobromine and CGA. Quantification is achieved by peak area measurement and comparison with standards.

### Genome and RNA sequencing

High-molecular-weight genomic DNA was extracted from fresh leaf tissue of *C. humblotiana* (accession RM-CF-00679). The ^plant conserved at the International Coffee Collection at La reunion was collected in 2010 in Mayotte (Mont Bénara). Long reads were obtained using PacBio RSII technology (63 SMRT cells). Forty-eight Gb of the sequences representing a coverage of about 102× (mean length 7.6 Kb) were generated. In addition, 24 Gb of 2 × 100 bp paired-end Illumina (Hiseq 2500) sequences (51 × coverage) were produced. The same tissue from the same genotype (RM-CF-00679) was also used to construct a library for Hi-C analysis and 90 million paired-end reads of 150 bp were obtained from the Illumina platform. In addition, RNA-seq was performed by extracting RNA from fresh leaves, and sequencing libraries were prepared using the Truseq Stranded Kit from Illumina according to the manufacture protocol. The libraries were sequenced using a HiSeq 2500 Illumina platform (2 × 150 bp) and 63,917,118 reads representing 9.5 Gb were generated. Genome quality reads and RNA Illumina reads were evaluated using FASTQC (https://www.bioinformatics.babraham.ac.uk/projects/fastqc/). Truseq sequence adapters were removed using Trimmomatic V.0.39^52^.

### Genome assembly

The size and heterozygosity of the *C. humblotiana* genome were estimated via the 21-mer depth distribution of Illumina reads. In detail, the k-mers were counted using Jellyfish^53^ and the k-mer count histogram was analyzed using Genoscope (http://qb.cshl.edu/genomescope/). A de novo genome assembly was performed using the FALCON assembler^54^. PBJelly^55^ was used for scaffolding and gap-closing the FALCON primary contigs and finally, assembly-polishing was performed using the Illumina data produced to correct potential errors in low-quality regions. Ninety-four percent of the Illumina reads map to the PBJelly2 contigs, 89% of those with the correct orientation and within the expected insert-size range. The assembly was performed by Computomics (https://computomics.com), while Hi-C was performed by Dovetail Genomics (https://dovetailgenomics.com). The quality of the genome assembly was estimated by searching for Benchmarking Universal Single-Copy Orthologs (BUSCO v4.0; https://busco.ezlab.org) with Embryophya odb 9. The *C. humblotiana* assembly was also evaluated for its contiguity by estimating the LTR Assembly Index (LAI) from LTR Retriever^26^. The genome-completement assessment was also conducted by mapping genome and RNA reads to the assembly using Bowtie2^27^ and Hisat2^28^.

### Genome annotation and gene prediction

The genome after-assembly and scaffolding with Hi-C was validated using a collinearity analysis with *C. canephora*. This revealed that some chromosomes needed to be reoriented to be in accordance with the chromosome orientation from *C. canephora*. This version of the genome was masked with RepeatMasker^56^ using a repeat library made with REPET from the *C. humblotiana* genome.

The trained models for AUGUSTUS^57^ for *C. humblotiana* were retrieved from BUSCO as it trains AUGUSTUS to achieve more precise results, using the mappings from its data set as a reference set. These files need minor intervention in order to make them generally useful for gene prediction. To achieve more precise prediction with AUGUSTUS, special care was taken to produce “hints”. These hints come from protein mapping and RNAseq produced in the frame of the project. The proteins were *C. canephora* proteins collected from NCBI, which were mapped with GenomeThreader^58^ (with parameters set as: − mincoverage 0.65 − minalignmentscore 0.7 − species Arabidopsis) onto the genome of *C. humblotiana*. The GenomeThreader output was converted into GFF3, and only CDS and intron features were kept as protein hints (source = P) for AUGUSTUS.

The RNAseq reads were cleared of any adaptors sequences and merged into longer SE reads based on their overlap (min 30 bp) into joined reads. Reads that did not comply with the requirements for joining were left as PE-reads. These reads were then mapped onto the genome using HISAT2 (-k 1 -no-unal -max-intronlen 35000 -min-intronlen 40) as single-end reads (also the remaining PE-reads). The resulting BAM file was processed with RegTools to extract junctions to bed format. This bed-file was converted into a GFF3 format and filtered for junctions with a minimum coverage of 10. From the resulting GFF3 file intron features were given as hints while the remaining exon bits on either side of the introns were given as an exon part (source = E). The accompanying parameter file for extrinsic data for AUGUSTUS was adapted to include these hints as well as to mask the genomic sequence. AUGUSTUS was run as augustus –species = $species –hintsfile = $hint –extrinsicCfgFile = extrinsic.MPE.cfg –softmasking = 1 $fasta.

The resulting gene predictions from AUGUSTUS were further curated with EvidenceModeler^59^ using the same extrinsic data. EVM managed this way to clean a few predicted genes with ambiguous structure and no support. The final predicted gene set (32,874) was subsequently processed to be uploaded into ORCAE (https://bioinformatics.psb.ugent.be/orcae/).

The proteomes from *C. canephora* (v 2014, 25,574), *Arabidopsis thaliana* (27,910) and the predicted gene set from *C. humblotiana* (32,874) were compared using OrthoFinder^60^ with default parameters.

Repeats were de novo identified using different approaches. First an ab initio identification was performed with REPET (V.2.5; https://urgi.versailles.inra.fr/Tools/REPET) leading to the identification of 430 consensuses after removing chimeric sequences and potential host genes. The final library comprises 3 helitrons (DHX), 168 DNA transposons (DTX), 79 unclassified transposons (DXX), 63 unclassified elements, 31 LINEs, 66 LTR retrotransposons, 6 SINEs, 14 unclassified retrotransposons. RepeatMasker was used with default parameters to mask the genomes and get statistics. LTR_STRUC^61^, LTR Retriever^26^ and Inpactor^62^ were used to recover and they classified full-length LTR retrotransposons. A circular annotation plot was performed using ShinyCircos^63^. A phylogenetic analysis of RT domains was performed as in Yu and coworkers^64^. The timing of LTR retrotransposon insertions was estimated as in Orozco-Arias et al.^62^ with full-length LTR retrotransposon recovered by LTR_STRUC and an average base substitution rate of 1.3E–8^29^.

### Comparative genome analysis

*Coffea canephora* and *C. humblotiana* genomes were globally compared using a dot plot as implemented in D-genies with the Minimap2 Aligner^31^ and i-ADHoRe^30^.

In order to retrieve the sequence containing the *S*_*H3*_ locus and the corresponding annotations from the *C. canephora* and *C. humblotiana* genomes, the region was manually reconstructed using five BAC clones belonging to each *Coffea arabica* sub-genome, as previously described^32^. Once reconstructed, the sequences were graphically aligned using Dotter^65^, and the common segment was extracted using the Extractseq function from the EMBOSS suite^66^ and then used to identify the equivalent coordinates on the *C. humblotiana* and *C. canephora* genomes using BLASTN^67^ with the default parameters. The region was extracted from each genome based on the coordinates found, using the Extractseq function, and the corresponding gene annotations were retrieved using the Bedtools intersect function^68^. Protein and CDS sequences from the region for each genome were also retrieved and were used to find orthologous gene pairs with the get_homologues-est.pl script from the Get_homologues program^69^, with the OrthoMCL option (-M). Additionally, a matrix of pairwise protein identity was obtained using the Clustal omega program^70^. Graphical comparisons between the two segments were completed using the Genoplotr R package.

### Phylogenetic analysis (NMT proteins)

Phylogenetic analyses of NMT genes were conducted using Seaview^71^. Proteins were aligned with Muscle and trees were computed using PhyML (Model LG, Branch support aLRT (SH-like); tree searching operation: NNI).

### NMT protein-sequence alignment and structural analysis

Sequence alignments were produced with Clustal Omega^70^ and ESPript 3.0^72^.

## Supplementary Information

Supplementary Information 1.

## Data Availability

Genome assembly information has been deposited at NCBI under the Bioproject ID: PRJNA665152. We built a *C. humbotiana* genome website at SGN (https://solgenomics.net) and ORCAE (https://bioinformatics.psb.ugent.be/orcae/), providing data download, Blast and genome browser. All data that support the findings of this study are also available from the corresponding authors upon request.
