# Supplementary material for: The absence of the caffeine synthase gene is involved in the naturally decaffeinated status of Coffea humblotiana, a wild species from Comoro archipelago
Source: Sci Rep. 2021 Apr 14;11:8119. doi: 10.1038/s41598-021-87419-0 (PMC8046976; doi:10.1038/s41598-021-87419-0)
Supplement: Supplementary file 1 — Supplementary Information 1. [file 41598_2021_87419_MOESM1_ESM.docx]

**The absence of the caffeine synthase gene is involved in the naturally decaffeinated status of *Coffea humblotiana,* a wild species from Comoro archipelago.**

Nathalie Raharimalala,^1 #^ Stephane Rombauts,^2,10 #^ Andrew McCarthy,^3^ Andréa Garavito,^4*^ Simon Orozco-Arias,^4,5^ Laurence Bellanger,^7^ Alexa Yadira Morales-Correa,^4^ Solène Froger,^7^ Stéphane Michaux,^7^ Victoria Berry,^7^ Sylviane Metairon ^6^, Coralie Fournier ^6,**^, Maud Lepelley,^7^ Lukas Mueller,^8^ Emmanuel Couturon,^9^ Perla Hamon,^9^ Jean-Jacques Rakotomalala,^1^ Patrick Descombes,^6^ Romain Guyot,^5,9^ *** Dominique Crouzillat.^7^ ***

**Supplementary Material (Tables, Figures and Data)**

**Supplementary Figure 1**

**A.** K-mer distribution with k=21 (K-mer number: 16,433,916,543) of sequencing Illumina reads from *C. humblotiana*. The peak position is at 36. **B.** Genomescope analysis with the same data


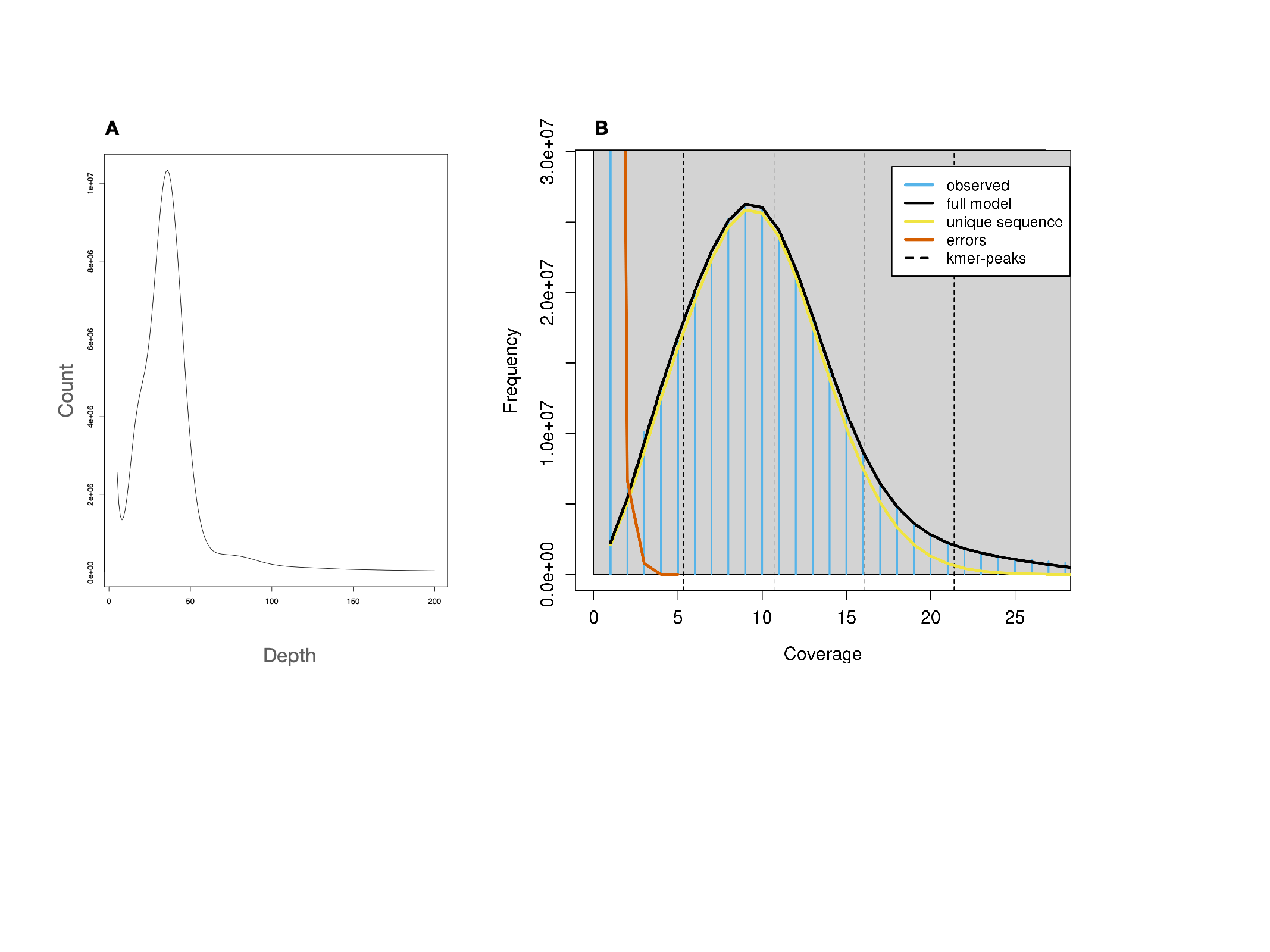


**Supplementary Table 1**

DNA- and RNA-read alignment rates against the final *C. humblotiana* assembly

| **Libraries** | **Library type** | **Sequence number** | **Overall alignment rate (Bowtie2)** | **Overall alignment rate (Hisat2)** |
| --- | --- | --- | --- | --- |
| **DNA HUMB1** | PE 2 X 100 bp | 2 X 51,900,860 | 86.7 % | - |
| **DNA HUMB2** | PE 2 X 100 bp | 2 X 67,786,173 | 86.51 % | - |
| **RNA HUMB1-1** | PE 2 X 150 bp | 2 X 7,089,600 | - | 88.62 % |
| **RNA HUMB1-2** | PE 2 X 150 bp | 2 X 7,075,448 | - | 88.65 % |
| **RNA HUMB2-1** | PE 2 X 150 bp | 2 X 8,903,758 | - | 86.87 % |
| **RNA HUMB2-2** | PE 2 X 150 bp | 2 X 8,889,753 | - | 86.85 % |

**Supplementary Table 2**

Predicted gene statistics of *C. humblotiana* and *C. canephora* (Denoeud et al., 2014).

|  | ***C. humblotiana*** | ***C. canephora**** |
| --- | --- | --- |
| Number of genes | 32,874 | 25,574 |
| Number of monoexonic genes | 7,291 | 5,004 |
| Number of multiexonic genes | 25,583 | 20,57 |
| Average overall gene size | 2,733.320 | 3,188.396 |
| Average overall CDS size | 1,000.579 | 1,205.554 |
| Average overall exon size | 214.368 | 236.219 |
| Average size of monoexonic genes | 650.244 | 963.384 |
| Median size of monoexonic genes | 441 | 711 |
| Largest monoexonic gene | 4,029 | 5,4 |
| Smallest monoexonic gene | 154 | 45 |
| Average size of multiexonic genes | 3,326.984 | 3,729.668 |
| Largest multiexonic gene | 45,815 | 219,753 |
| Smallest multiexonic gene | 241 | 84 |
| Average size of multiexonic exons | 192.623 | 207.228 |
| Average size of multiexonic introns | 472.448 | 483.203 |
| Average number of exons per multiexonic gene | 5.713 | 6.102 |
| Largest multiexonic exon | 5,993 | 6,574 |
| Smallest multiexonic exon | 3 | 6 |
| Most exons in one gene | 62 | 65 |
| Average number of introns per multiexonic gene | 4.713 | 5.102 |
| Median number of introns per multiexonic gene | 3 | 4 |
| Largest intron | 12,721 | 216,594 |
| Smallest intron | 42 | 9 |

**Supplementary Table 3**

List of the accessions used for biochemical analysis. *C. arabica* genotypes were coming from wild (ET39) and cultivated accessions GPFA03 (catura) and hybrids (wild Ethiopian x catimor). For *C. canephora,* we selected the accessions according to genetic groups (Merot et al., 2019) with one conilon genotype (cultivated in Brazil), and two hybrids between different genetic groups. *C. humblotiana* plantlets were from seeds of the same population (open pollinated) than the sequenced genotype (Origin Mayotte). These plants are not available anymore due the destructive process of biochemical analyses on young plantlets.

| **Accessions** | **Species** | **Geographic origins** | **Living Collection** |
| --- | --- | --- | --- |
| 16M | *C. canephora Pierre ex A.Froehner* | West Africa | Nestlé Tours |
| FRT95 | *C. canephora Pierre ex A.Froehner* | West Africa | Nestlé Tours |
| FRT141-8 | *C. canephora Pierre ex A.Froehner* | West Africa | Nestlé Tours |
| GPFA03 | *C. arabica L.* | Ethiopia | Nestlé Tours |
| ET39 | *C. arabica L.* | Ethiopia | Nestlé Tours |
| GPFA107 | *C. arabica L.* | Ethiopia | Nestlé Tours |
| OP1 | *C. humblotiana Baill.* | Mayotte | - |
| OP2 | *C. humblotiana Baill.* | Mayotte | - |
| OP3 | *C. humblotiana Baill.* | Mayotte | - |

**Supplementary Figure 2**

Venn Diagram of the Orthofinder comparison between the proteomes from *C. canephora* (v 2014, 25,574), Arabidopsis (27,910) and the predicted gene set from *C. humblotiana* (32,874).

*C. humblotiana*

*C. canephora*

*Arabidopsis thaliana*

16,350 gene families

25

486

11382

(7021)

283

52

11

4111

**Supplementary Figure 3**

Comparison of transposable elements content between *C. canephora* and *C. humblotiana* (in percentage of genome sequence size).

**Supplementary Figure 4**

Phylogenetic analysis of *C. humblotiana* Reverse Transcriptase (RT) domains from LTR retrotransposons. The unrooted phylogenetic tree of Gypsy and Copia elements was constructed based on 1,992 aligned reverse transcriptases (minimum of 200 residues).


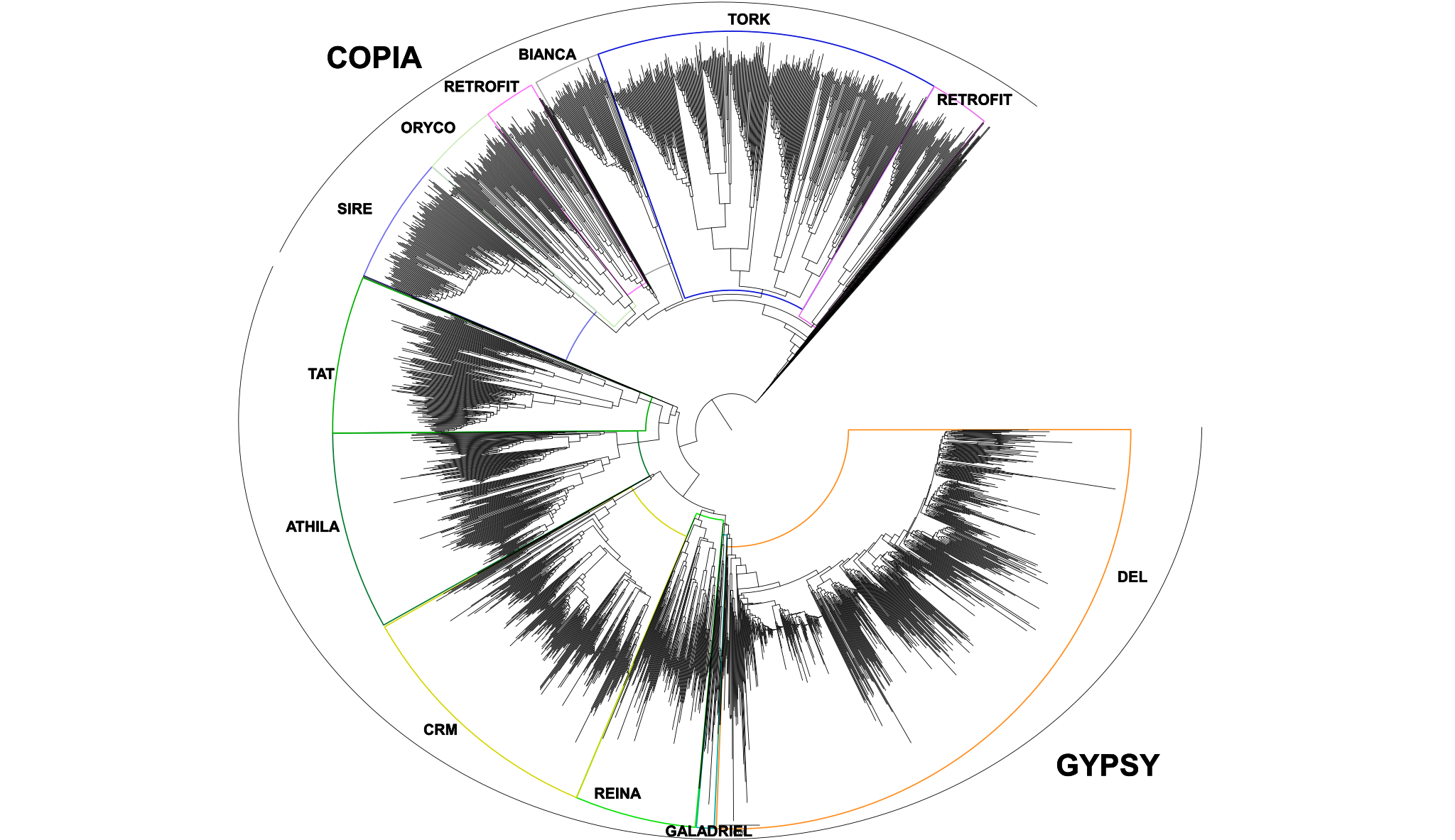


**Supplementary Figure 5**

Phylogenetic analysis of *C. humblotiana* and C. canephora Reverse Transcriptase (RT) domains from LTR retrotransposons. Branches in blue underline specific amplification of elements in *C. canephora* while branches in orange show those in *C. humblotiana*.


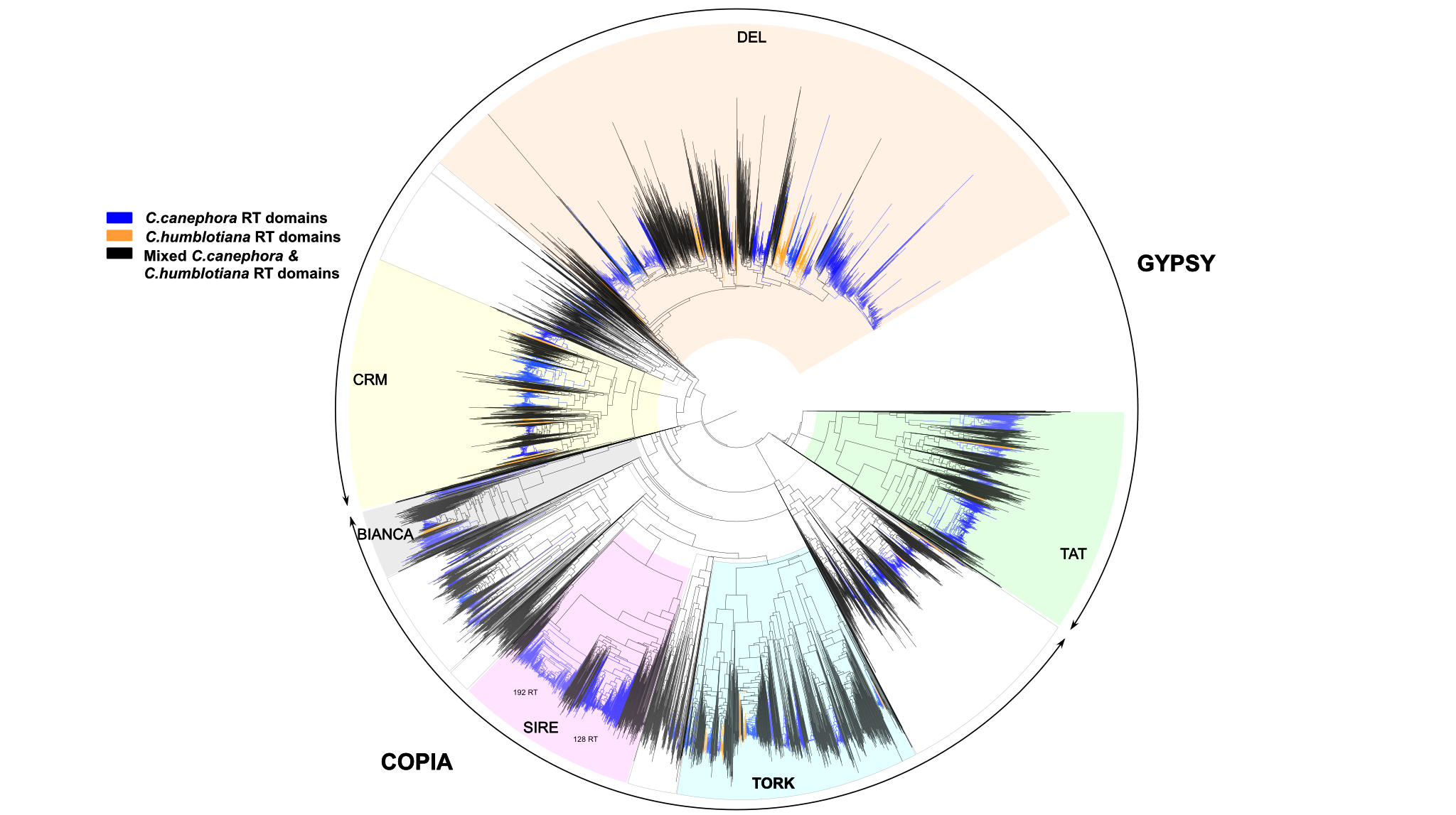


**Supplementary Figure 6**

Comparison of TE dynamics between *C. canephora* and *C. humblotiana*. A. Timing of all LTR Retrotransposon insertions in *C. canephora* and *C. humblotiana*. Full-length LTR retrotransposons identified by LTR_STRUC per bins of 0.5 MY. An average base substitution rate of 1.3E–8 was used (Ma and Bennetzen 2004). B. Timing of LTR Retrotransposon lineage insertions in *C. canephora*. C. Timing of LTR Retrotransposon lineage insertions in *C. humblotiana.*

**Supplementary Figure 7**

Comparative structural genomics between *C. humblotiana and C. canephora.*

A. Whole genome dot-plot between *C. canephora* and *C. humblotiana* using Symap (https://github.com/csoderlund/SyMAP). 0 indicates the unplaced contigs of *C. canephora*. B. Circular representation of the syntenic blocks between *C. humblotiana and C. canephora* using Symap.

**Supplementary Figure 8**

Dot plot between *C. canephora* (CC) and *G. jasminoides* (Gj) at the DXMT locus on Chromosome 1. Representation of the microsynteny between *C. canephora* (CC; Chromosome 1 position 1-1.4 Mb) and *G. jasminoides* (Gj; Chromosome 5 position 2.2-2.5 Mb). The duplicated regions flanking the DXMT locus in CC (grey boxes) are represented by black boxes for CC and Gj.


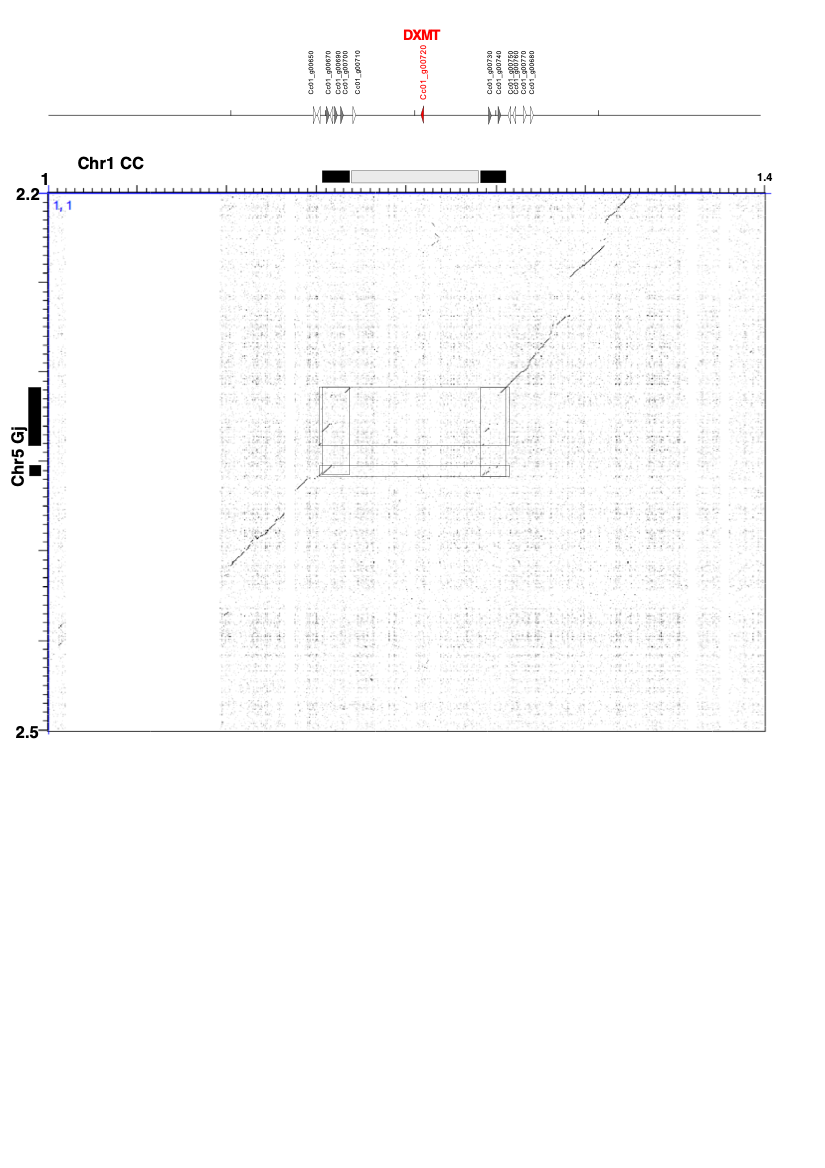


**Supplementary Figure 9**

Microsynteny between *C. canephora* (CC) and *C. humblotiana* (CH) at the NMT2 locus on Chromosome 2. Representation of the microsynteny between *C. canephora* (CC; Chromosome 2 position 7.2-7.4 Mb) and *C. humblotiana* (CH; Chromosome 2 position 7.5-7.7 Mb). The NMT2 genes are indicated in red. Lines indicate the conservation of genes named by their ID.

**
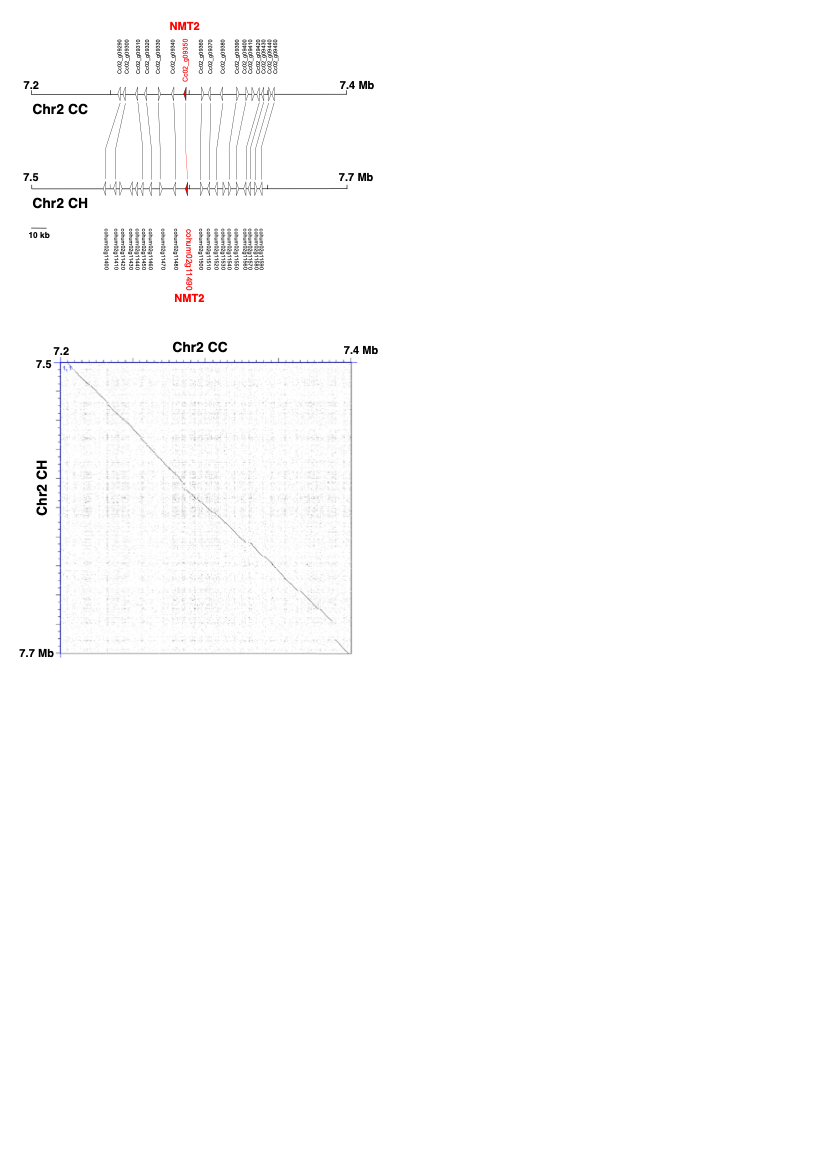
**

**Supplementary Figure 10**

Dot plot between *C. humblotiana* (CH) and *G. jasminoides* (Gj) at the NMT2 locus on Chromosome 2. Representation of the microsyntheny between *C. humblotiana* (CH; Chromosome 2 position 7.5-7.7 Mb) and *G. jasminoides* (Gj; Chromosome 9 position 103.2-103.3 Mb).


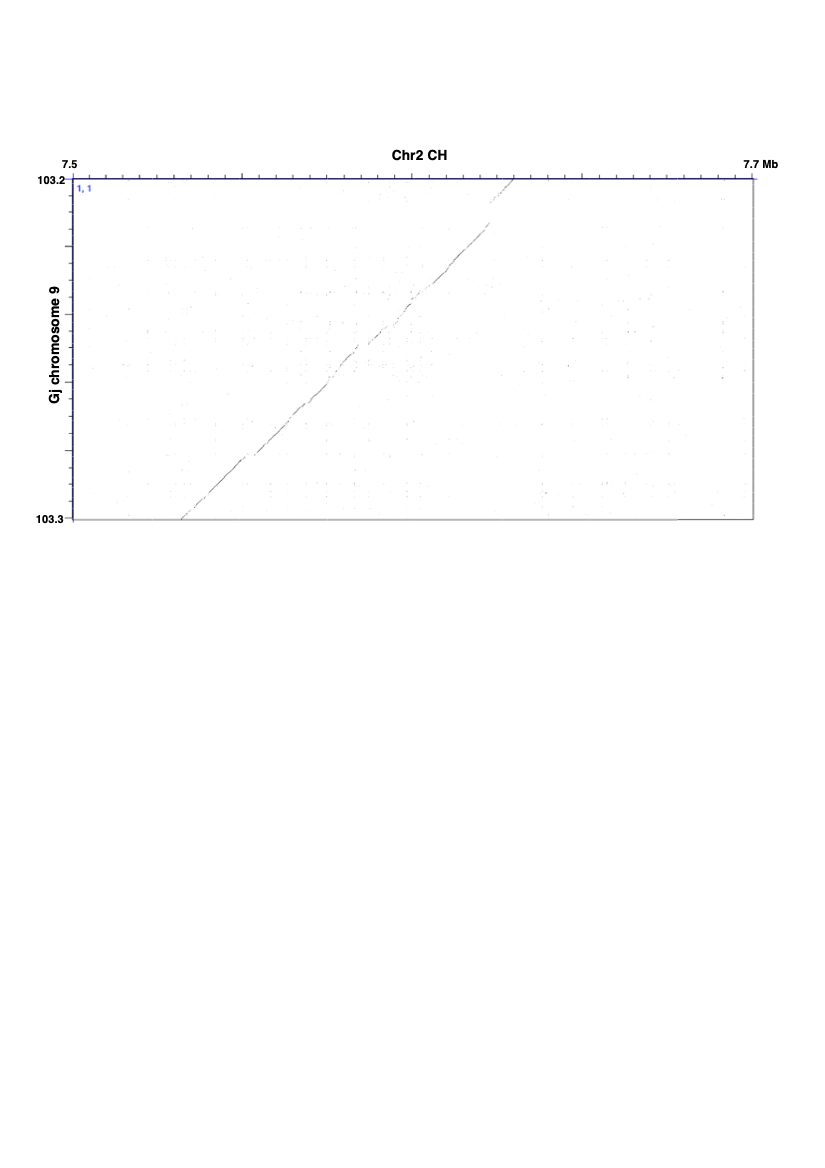


**Supplementary Data 1**

FASTA sequences of NMT proteins used in our study from *C. canephora* (Cc, Denoeud et al., 2014; Perrois et al., 2015), *C. humblotiana* (cohum) and *G. jasminoides* (GJ).

>Cc00_g24720_1 Monomethylxanthine methyltransferase 1

MELQEVLHMNEGEGDTSYAKNASYNLALAKVKPFLEQCIRELLRANLPNINKCIKVADLG

CASGPNTLLTVRDIVQSIDKVGQEEKNELERPTIQIFLNDLFQNDFNSVFKLLPSFYRKL

EKENGRKIGSCLISAMPGSFYGRLFPEESMHFLHSCYSVHWLSQVPSGLVIELGIGANKG

SIYSSKGCRPPVQKAYLDQFTKDFTTFLRIHSKELFSRGRMLLTCICKVDEFDEPNPLDL

LDMAINDLIVEGLLEEEKLDSFNIPFFTPSAEEVKCIVEEEGSCEILYLETFKAHYDAAF

SIDDDYPVTSHEQIKAEYVASLIRSVYEPILASHFGEAIMPDLFHRLAKHAAKVLHMGKG

CYNNLIISLAKKPEKSDV*

>Cc01_g00720_1 3,7-dimethylxanthine N-methyltransferase

MELQEVLHMNGGEGDTSYAKNSSYNLFLIRVKPVLEQCIQELLRANLPNINKCFKVGDLG

CASGPNTFSTVRDIVQSIDKVGQEKKNELERPTIQIFLNDLFQNDFNSVFKLLPSFYRNL

EKENGRKIGSCLIGAMPGSFYSRLFPEESMHFLHSCYCLHWLSQVPSGLVTELGISVNKG

CIYSSKASRPPIQKAYLDQFTKDFTTFLRIHSEELISRGRMLLTFICKEDEFDHPNSMDL

LEMSINDLVVEGHLEEEKLDSFNVPIYAPSTEEVKRIVEEEGSFEILYLETFYAPYDAGF

SIDDDYQGRSHSPVSCDEHARAAHVASVVRSIYEPILASHFGEAILPDLSHRIAKNAAKV

LRSGKGFYDSVIISLAKKPEKADM*

>Cc02_g09350_1 Probable caffeine synthase 3

MELQRVLHMSGGEGDTSYAKNSSYQVKPVLEQCIQELLRTNLPYDEKCIRVADLGCSSGP

NTLLTVSDIIQSIDKVSQEMDNEFALPTIQVFLNDLFENDFNTVIKSLPSFYRKLEKENG

RKIGSCLIAAMPGSFYGRLFPEQSVHFLHSSYSLHWLSQVPNGLVTESGISANKGSIYSS

KASPPAIQKAYLDQFTKDFTTFLRMHSEELVSHGRILLTFMCKGDEFDGPNILDLLEVAI

NDLVVEGSLEEEKLDSFNVPIYAPSVEEVRHIIEEERSFEIVYLETFKLRHDAGFSIDDN

QAAHVASFVRAAWEPILASHFGEAIIADLFHRFAKNAATPLRMGKGFFNNLIISLAKKPH

KSETCKYLFLDM*

>Cc09_g06950_1 Probable caffeine synthase 4

MELQEVLHMNGGEGEASYAKNSSFNQLVLAKVKPVLEQCVRELLRANLPNINKCIKVADL

GCASGPNTLLTVRDTVQSIDKVRQEMKNELERPTIQVFLTDLFQNDFNSVFMLLPSFYRK

LEKENGRKIGSCLIAAMPGSFHGRLFPEESMHFLHSSYSLQFLSQVPSGLVTELGITANK

RSIYSSKASPPPVQKAYLDQFTKDFTTFLRMRSEELLSRGRMLLTCICKGDECDGPNTMD

LLEMAINDLVVEGRLGEEKLDSFNVPIYTASVEEVKCMVEEEGSFEILYLQTFKLRYDAG

FSIDDDCQVRSHSPEYSDEHARAAHVASLIRSVYEPILASHFGEAIIPDIFHRFATNAAK

VIRLGKGFYNNLIISLAKKPEKSDI*

>Cc09_g06960_1 Theobromine synthase 2

MELQEVLHMNGGEGDTSYAKNSSYNQLVLTKVKPVLEQCIRELLRANLPNINKCIKVADL

GCASGPNTLLTVRDIVQSIDKVGQEEKNELEHPTIQIFLNDLFQNDFNSVFKLLPSFYRK

LEKENGRKIGSCLISAMPGSFYGRLFPEESMHFLHSCYSVHWLSQVPSGLVTELGISANK

GIIYSSKASPPPVQKAYLDQFTKDFTTFLRIHSEELLSGGRMLLTCICKGDESDGLNTID

LLERAINDLVVEGLLEEEKLDSFNLPLYTPSLEVVKCIVEEEGSFEILYLETFKVRYDAG

FSIDDDYQVRSLFQVYCDEHVKAAYVTFFFRAVFEPILASHFGEAIMPDLFHRFAKNAAK

ALRLGNGFYNSLIISLAKKPEKSDM*

>Cc09_g06970_1 7-methylxanthosine synthase 1

MELQEVLRMNGGEGDTSYAKNSAYNQLVLAKVKPVLEQCVRELLRANLPNINKCIKVADL

GCASGPNTLLTVRDIVQSIDKVGQEKKNELERPTIQIFLNDLFPNDFNSVFKLLPSFYRK

LEKENGRKIGSCLIGAMPGSFYSRLFPEESMHFLHSCYCLQWLSQVPSGLVTESGISTNK

GSIYSSKASRLPVQKAYLDQFTKDFTTFLRIHSEELFSHGRMLLTCICKGVELDARNAID

LLEMAINDLVVEGHLEEEKLDSFNLPVYIPSAEEVKCIVEEEGSFEILYLETFKVLYDAG

FSIDDEHIKAEYVASSVRAVYEPILASHFGEAIIPDIFHRFAKHAAKVLPLGKGFYNNLI

ISLAKKPEKSDV*

>Cc09_g07000_1 3,7-dimethylxanthine N-methyltransferase

MELQHILHMNGGEGDTSYAKNSSYQNLVLTKAKPVLERCMRELLPANLPNINKCIKVADL

GCSSGPNTLLTAWNIIQSIDKVGQEKKNALEPPTIQIFLNDLFQNDFNSVFKSLPSFYRK

LEKENGRKIGSCQIAAMPGSFYGRLFPEESMHFLHSSYGLHWLSQVPSGLVTESGISVNK

GSIYSSKASCPPAQKAYLDQFMKDFTTFLRMHSELGTVGFTWPNTPYFHV*

>ccdxmt-JX978516 AFV60444.1 3,7-dimethylxanthine methyltransferase [Coffea canephora]

MELQEVLHMNGGEGDTSYAKNSSYNLFLIRVKPVLEQCIQELLRANLPNINKCFKVGDLG

CASGPNTFSTVRDIVQSIDKVGQEKKNELERPTIQIFLNDLFQNDFNSVFKLLPSFYRNL

EKENGRKIGSCLIGAMPGSFYSRLFPEESMHFLHSCYCLHWLSQVPSGLVTELGISVNKG

CIYSSKASRPPIQKAYLDQFTKDFTTFLRIHSEELISRGRMLLTFICKEDEFDHPNSMDL

LEMSINDLVVEGHLEEEKLDSFNVPIYAPSTEEVKRIVEEEGSFEILYLETFYAPYDAGF

SIDDDYQGRSHSPVSCDEHARAAHVASVVRSIYEPILASHFGEAILPDLSHRIAKNAAKV

LRSGKGFYDSVIISLAKKPEKSDV

>ccMXMT1-jx978517 AFV60445.1 7-methylxanthine methyltransferase 1 [Coffea canephora]

MELQEVLHMNEGEGDTSYAKNASYNLALAKVKPFLEQCIRELLRANLPNINKCIKVADLG

CASGPNTLLTVRDIVQSIDKVGQEEKNELERPTIQIFLNDLFQNDFNSVFKLLPSFYRKL

EKENGRKIGSCLISAMPGSFYGRLFPEESMHFLHSCYSVHWLSQVPSGLVIELGIGANKG

SIYSSKGCRPPVQKAYLDQFTKDFTTFLRIHSKELFSRGRMLLTCICKVDEFDEPNPLDL

LDMAINDLIVEGLLEEEKLDSFNIPFFTPSAEEVKCIVEEEGSCEILYLETFKAHYDAAF

SIDDDYPVTSHEQIKAEYVASLIRSVYEPILASHFGEAIMPDLFHRLAKHAAKVLHMGKG

CYNNLIISLAKKPEKSDV

>ccXMT1 sp|A4GE69.2|XMT1_COFCA RecName: Full=7-methylxanthosine synthase 1; AltName: Full=Xanthosine methyltransferase; Short=XMT

MELQEVLRMNGGEGDTSYAKNSAYNQLVLAKVKPVLEQCVRELLRANLPNINKCIKVADL

GCASGPNTLLTVRDIVQSIDKVGQEKKNELERPTIQIFLNDLFPNDFNSVFKLLPSFYRK

LEKENGRKIGSCLIGAMPGSFYSRLFPEESMHFLHSCYCLQWLSQVPSGLVTESGISTNK

GSIYSSKASRLPVQKAYLDQFTKDFTTFLRIHSEELFSHGRMLLTCICKGVELDARNAID

LLEMAINDLVVEGHLEEEKLDSFNLPVYIPSAEEVKCIVEEEGSFEILYLETFKVLYDAG

FSIDDEHIKAEYVASSVRAVYEPILASHFGEAIIPDIFHRFAKHAAKVLPLGKGFYNNLI

ISLAKKPEKSDV

>cohum02g11490

MELQRVLHMSGGEGDTSYAKNSSYQKLVLTKVKPVLDQCIQELLRTNLPYNKKCIRVADL

GCSSGPSTLLTVSDIIQSIDKVSQEMDNEFALPTIQVFLNDLFENDFNTVIKSLPSFYHK

LEKENGRKIGSRLIAAMPGSFYGRLFPEQSIHFLHSSYSLHWLSQVPSGLVTEPGISANK

GSIYSSKASRPAIQKAYLDQFTKDFTTFLRMHSEELGRLEEEKLDSFNVPIYTPSVEEVR

HIIEEEGSFEIVYLETFKLRHDAGFSIDDDQLGSHSQVHFCDQHVRAAHVASFIRAVSEP

ILASHFGEAIIPVLFHRFTKNAAKLLRMGKGFFNNLIISLAKKPHKSDM

>cohum09g08730

MEAKAMQATPRIHPSIKLVLAKVKPVLEQCIRELLRANLPNINKCIKVADLGCASGPNTL

LTVRDIVRSIDKVRQEMKNELERPTIQVFLTDLFQNDFNSVFMLLPSFYRKLEKENGRKI

GSCLIAAMPGSFHGRLFPDESMHFLHSSYSLHFLSQVPSGLVTELGITANRRSIYSSKAS

PPPVQKAYLDQFTKDFTAFLRIRSEELLSRGRMLLTCICKGDEFDGPNTMDLLEMAINDL

VVEGHLEEEKLDSFNVPIYTTSVEELKCIVEEEGSFEILFLETFKLRYDAGFSIDDDCQV

RSHSPVYSDEHARAAHVASLIRSVYEPILASHFGEAVIPDIFHRFATNAAKVIRLGKGFY

NNLVISLAKKPEKSDI

>cohum09g08760

MELQAVLHMNGGEGDTSYAKNSSYNQLALAKVKPVLEQCIRELLRANLPNINNCIKVADL

GCASGPNTLLTVRDIVQSIDKFGQEEKNELERPTIQIFLNDLFQNDFNSVFKLLPSFYRK

LEKENGRKIGSCLISAMPGSFYGRLFPEESMHFLHSCYSFHWLSQVPSGLVIELGISANK

GSIYSSKASRPPVQKAYLDQFTKDFATFLRIHSKELFSRGRMLLTCICKVDEFDEPNPLD

LLDMAINDLIVEGHLEEEKLASFNLPFFTPSAEEVKCIVEEEGSFEILYLETFKAHYDAG

FSIDDDYPVRSHFQVYCDEHIKAEYVASLIRSVYEPILASHFGEAIMPDLFHRLAKHAAK

VLHLGKGCYNNLIISLAKKPEKSDM

>cohum09g08800

MELQAVLHMNGGEGDTSYAKNSAYNHLVLNKVKPVLEQCIRELLRANLPNINKCIKVADL

GCASGPNTLLTVRDIVRSIDKVGQEKNELERRTIQIFLNDLFQNDFNSVFKLLPSFYSKL

EQENGRKIGSCLIGAMPGSFYDRLFPAESMHFLHSCYSLHWLSQVPSGLVTELGISTNKG

SIYSSKASRPPVQKAYLDQFTKDFTTFLRIHSEELFSRGRMLLTCICKGVEFDALNAIDL

LEMAINDLVVEGHLEEEKLDSFNLPVYIPSAEEVKCIVEEEGSFEILYLETFKVLYDAGF

SIDDDYPVRSHVQVYRDEHIKAEYVASSVRAVYEPILASHFGEAIIPDIFHRFAKHAAKV

LPLGKGFYNNLIISLAKKPEKPDM

>cohum09g08820

MELQEVLHMNGGEGDTSYAKNSSYNQLVLTKVKPVLEQCIRELLRANLPNINKCIKVADL

GCGSGPNTLLTVRDIVQSVDKVRQEMKNELEHPTIQIFLNDLFQNDFNSVFKLLPSFYRK

LEKENGRKIGSCLIAAMPGSFYSRLFPEESMHFLHSCYSLHWLSQVPSGLVTELGISANK

GIIYSSKASPPPVQKAYLDQFTKDFTTFLSIHSEELLSRGRMLLTCICKGDESDGLNTID

LLERAINDLVVEGLLEEENLDSFNLPLYTPSLEVVKCIVEEEGSFEILYLETFKVRYDAG

FSIDDDYQVRSHFQVYCDEHVKAAYVASFVRAVFEPIILEKLLYLTYSTGLRRMQQRFSA

WAAASIIVLSFLLPKNQRSQTCKSLFLVGFCVVVCFQICGKDLVRMGFYPGIVLFILVGV

>cohum09g08830

MELQEVLHMNGGEGDTSYAKNSSYNQLVLTKVKPVLEQCIRELLRANLPNINKCIKVADL

GCGSGPNTLLTVRDIVQSVDKVRQEMKNELEHPTIQIFLNDLFQNDFNSVFKLLPSFYRK

LEKENGRKIGSCLIAAMPGSFYSRLFPEESMHFLHSCYSLHWLSQVPSGLVTELGISANK

GIIYSSKASPPPVQKAYLDQFTKDFTTFLRIHLEELLSRGRMLLTCICKGDESDGLNTID

LLERAINDLVVEKAAYVASFVRAVFEPILASHFGEAIIPDLFHRFAKNAAKVLRLGNGFY

NSLVISLAKKPEKSDM

>GJ9

MELQQVLHMSGGEGDTSYFKNSSYQVWLVLTKVKPVLEQCIQELLRTNLQPYNKKCIRVA

GLGCSSGPNTLLTVSDIIQSIDKVCQEMDDEFVPPTIQVFLNDLFENDFNTVIRSLPSFY

NKLETENGRKMGSCLIAAMPGSFYGRLFPQQSMHFLHSSNGLQWLSQVPSGLVTESGISA

NKGSIYSSKASRPPVQKAYLDQFMKDFTAFLRMHSEELVSGGRMLLTFVCKGDEFDGPNI

LDLLEMAVNDLVVEGHLEEEKLDSFNVPIYTPSVEEVRYTVEEEGSFEIVYLETFKLRHD

AGFCIDDDSRLGSHSQVYRDEHVRAAHVASFTRAALEPILASHFGEAIIPDLFHRFAKNA

AEVLRMGKRFANNIIISLAKKPVRSD
